# Supplementary material for: The Nucleocapsid Protein of SARS-CoV-2, Combined with ODN-39M, Is a Potential Component for an Intranasal Bivalent Vaccine with Broader Functionality
Source: Viruses. 2024 Mar 8;16(3):418. doi: 10.3390/v16030418 (PMC10976088; doi:10.3390/v16030418)
Supplement: Supplementary file 1 [file viruses-16-00418-s001.zip › viruses-2877407-supplementary.pdf]

Supplementary Figures

Captions:

Supplementary Figure S1. Antibody response against N (Delta strain) protein measured by ELISA in mice samples. IgG in sera (A), and IgA in BALF (B). The samples were collected 15 days after the administration of two and three doses of N+ODN-39M by intranasal route. Data are expressed as Log titers (A) and O.D 492nm (B), the horizontal line represents the mean.

Supplementary Figure S2. IgG1/IgG2a ratios were determined from quantitative measurements of anti-N-specific serum IgG1 and IgG2a measured, on individual mice, 12 days after the third immunization. Six- to 8-week-old mice were immunized with three doses of each formulation, at days 0, 7 and 21, according to the following group design: G1: N+Alum, sc. G2: N+ODN-39M+Alum, sc. G3: N (PBS), in. G4: N+ODN-39M, in. G5: PBS+Alum, sc. G6: PBS, in. sc: subcutaneous, in: intranasal.

Supplementary Figure S3. Mucosal humoral immune response measured in bronchoalveolar lavage fluid (BALF) by IgG ELISA against N, twelve days after the third immunization. Six- to 8-week-old mice were immunized with three doses of each formulation, at days 0, 7 and 21, according to the following group design: G1: N+Alum, sc. G2: N+ODN-39M+Alum, sc. G3: N (PBS), in. G4: N+ODN-39M, in. G5: PBS+Alum, sc. G6: PBS, in. sc: subcutaneous, in: intranasal. Data are expressed as O.D, the horizontal line represents the mean.

Supplementary Figure S4. IgG against different N proteins measured by ELISA in mice sera (dilution 1:1000) after the third immunization with N+ODN-39M by intranasal route. Data are expressed as O.D, the horizontal line represents the mean.

Supplementary Figure S5. IFN-γ spleen secreting cells response by ELISPOT against different stimulating agents (N351-365 peptide, and N proteins from SARS-CoV-1, MERS-CoV, and HCoV-229E) measured 26 days after the third immunization in mice immunized with N+ODN-39M (in). The empty square symbol in each case represents the result generated by a pool of three placebo immunized mice. The horizontal line represents the mean.

Supplementary Figure S6. IgG response in sera (dilution 1:1000) against N proteins from SARS-CoV-2 Delta strain and SARS-CoV-1. The ELISA was carried out with samples collected after three doses from mice intranasally immunized with N+ODN-39M+RBD. Data are expressed as O.D, the horizontal line represents the mean.

Supplementary Figure S7. IgG response against RBD protein from SARS-CoV-2 Omicron strain. The antibodies were measured by ELISA in sera, twelve days after the third dose in mice immunized intranasally with N+ODN-39M+RBD, N+RBD, RBD, and PBS. The horizontal line represents the mean.

Figure S1.

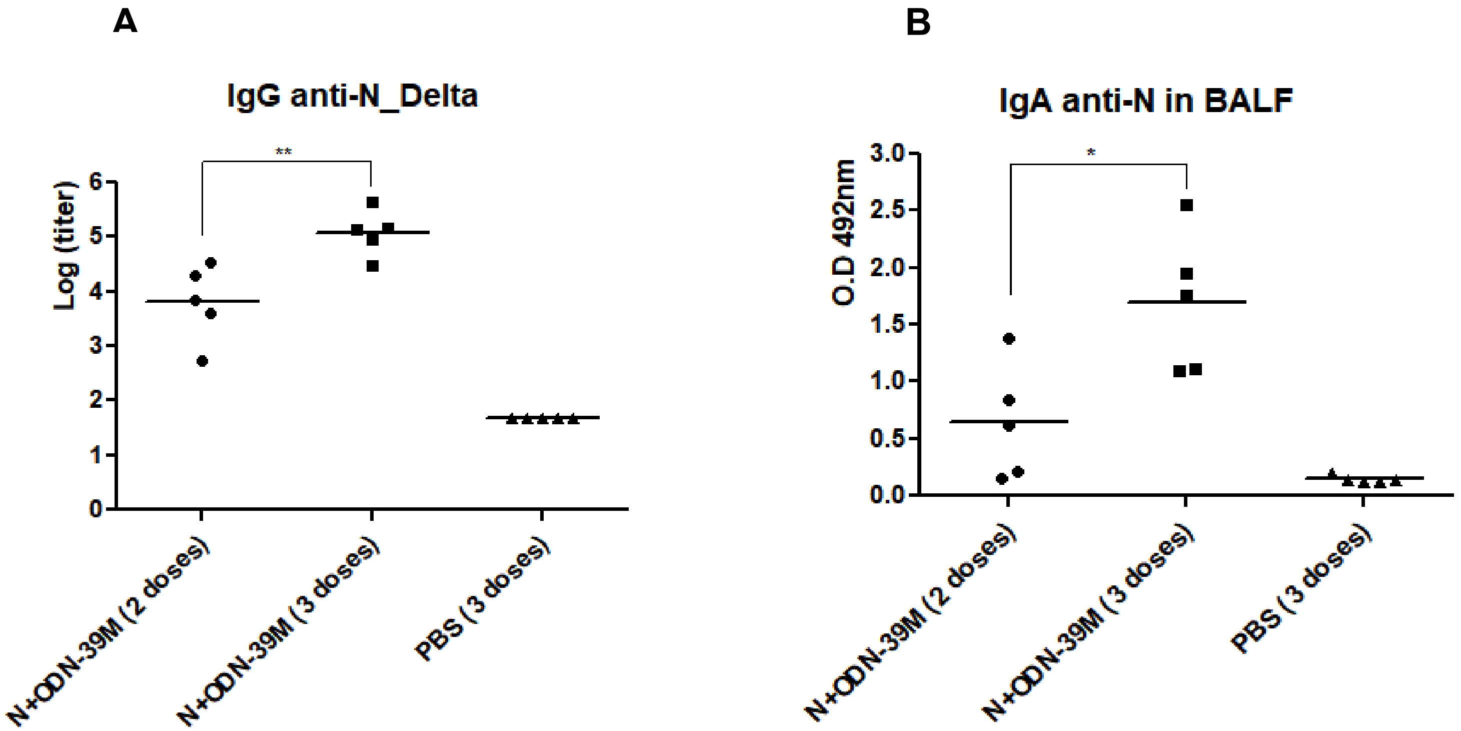

A
IgG anti-N_Delta
**
Log (titer)
0
1
2
3
4
5
6
N+ODN-39M (2 doses)
N+ODN-39M (3 doses)
PBS (3 doses)
B
IgA anti-N in BALF
*
O.D 492nm
0.0
0.5
1.0
1.5
2.0
2.5
3.0
N+ODN-39M (2 doses)
N+ODN-39M (3 doses)
PBS (3 doses)

Figure S2

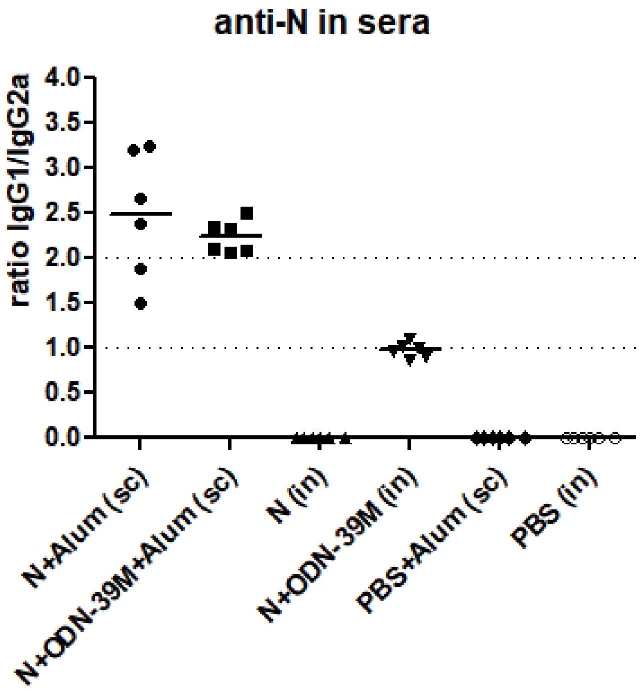
anti-N in sera
ratio IgG1/IgG2a
4.0
3.5
3.0
2.5
2.0
1.5
1.0
0.5
0.0
N+Alum (sc)
N+ODN-39M+Alum (sc)
N (in)
N+ODN-39M (in)
PBS+Alum (sc)
PBS (in)

Figure S3.

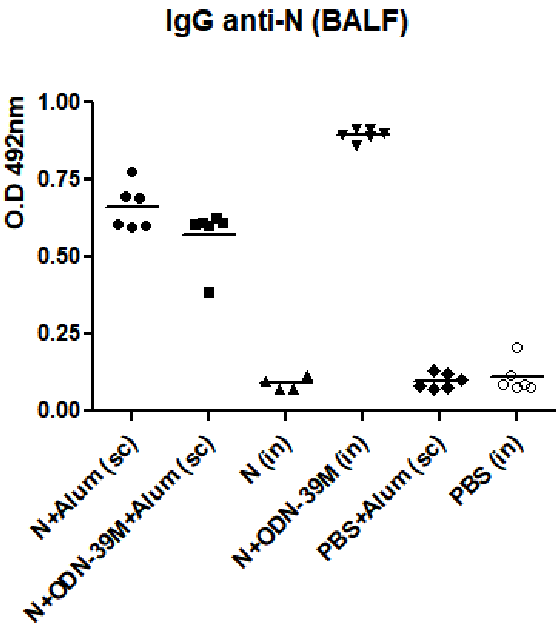
IgG anti-N (BALF)
O.D 492nm
1.00
0.75
0.50
0.25
0.00
N+Alum (sc)
N+ODN-39M+Alum (sc)
N (in)
N+ODN-39M (in)
PBS+Alum (sc)
PBS (in)

Figure S4.

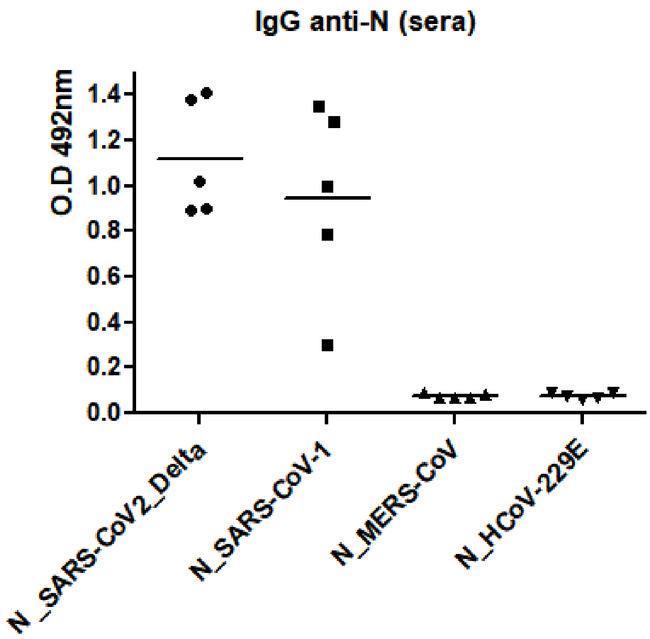

IgG anti-N (sera)
O.D 492nm
1.4
1.2
1.0
0.8
0.6
0.4
0.2
0.0
N _SARS-CoV2_Delta
N_SARS-CoV-1
N_MERS-CoV
N_HCoV-229E

Figure S5

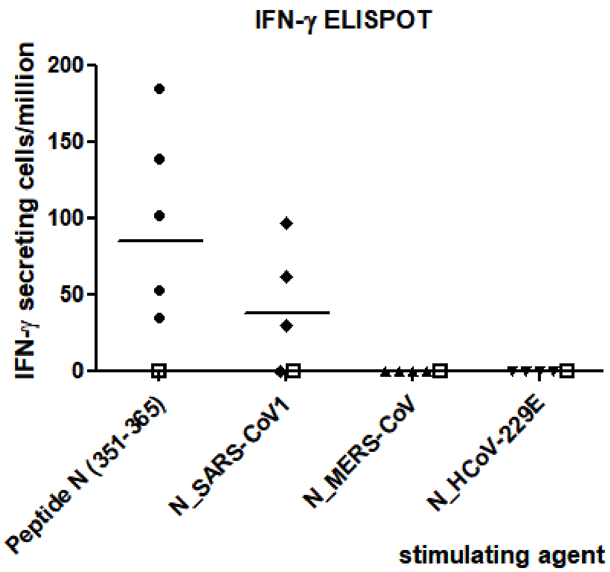

IFN-γ ELISPOT
IFN-γ secreting cells/million
200
150
100
50
0
Peptide N (351-365)
N_SARS-CoV1
N_MERS-CoV
N_HCoV-229E
stimulating agent

Figure S6.

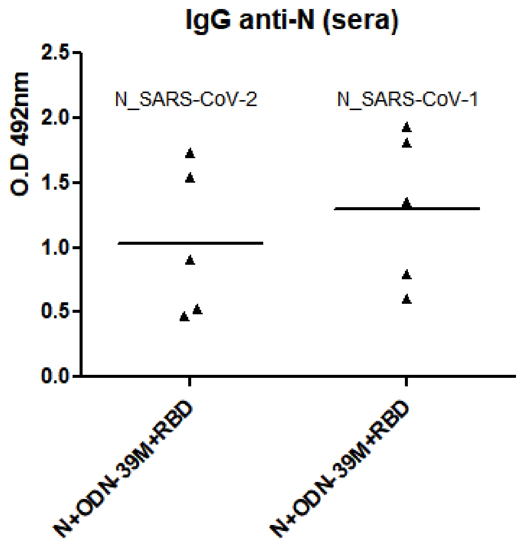

IgG anti-N (sera)
2.5
2.0
1.5
1.0
0.5
0.0
O.D 492nm
N_SARS-CoV-2
N_SARS-CoV-1
N+ODN-39M+RBD
N+ODN-39M+RBD

Figure S7.

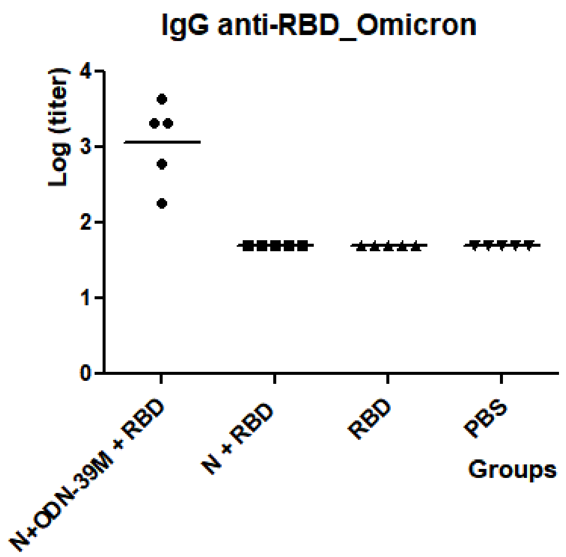
IgG anti-RBD_Omicron
Log (titer)
0
1
2
3
4
N+ODN-39M + RBD
N + RBD
RBD
PBS
Groups
